# Donation Parties

**Published:** 1876-04

**Authors:** 


Surprise-visits, lottery-drawings, fairs, *et id omne*, away with them! they are a hypocrisy, a cheat, a degradation, the whole of them, not a single exception, and being so, the sooner they are abandoned the better.

Donation-parties and surprise-visits are the ways and means of giving material aid to clergymen, who either need it or do not: if they do not need this aid, then the proceedings are simply a stultification of all concerned; if they do need such aid, it shows the great inconsideration if not actual injustice, of those to whom the minister preaches; it clearly indicates the fact that he is not properly sustained, and that his parishioners know it.

